# Cortical Neurovascular Coupling Driven by Stimulation of Channelrhodopsin-2

**DOI:** 10.1371/journal.pone.0046607

**Published:** 2012-09-28

**Authors:** Lijun Ji, Junli Zhou, Rabia Zafar, Svetlana Kantorovich, Ruixin Jiang, Paul R. Carney, Huabei Jiang

**Affiliations:** 1 The J. Crayton Pruitt Family Department of Biomedical Engineering, University of Florida, Gainesville, Florida, United States of America; 2 Department of Pediatrics, University of Florida, Gainesville, Florida, United States of America; 3 Department of Neurology, University of Florida, Gainesville, Florida, United States of America; 4 McKnight Brain Institute, University of Florida, Gainesville, Florida, United States of America; 5 B. J. and Eve Wilder Center for Excellence in Epilepsy Research, University of Florida, Gainesville, Florida, United States of America; Instituto de Neurociencias de Alicante UMH-CSIC, Spain

## Abstract

While functional imaging is widely used in studies of the brain, how well the hemodynamic signal represents the underlying neural activity is still unclear. And there is a debate on whether hemodynamic signal is more tightly related to synaptic activity or action potentials. This study intends to address these questions by examining neurovascular coupling driven by pyramidal cells in the motor cortex of rats. Pyramidal cells in the motor cortex of rats were selectively transduced with the light sensitive cation channel channelrhodopsin-2 (ChR2). Electrophysiological recordings and optical intrinsic signal imaging were performed simultaneously and synchronously to capture the neural activity and hemodynamics induced by optical stimulation of ChR2-expressing pyramidal cells. Our results indicate that both synaptic activity (local field potential, LFP) and action potentials (multi-unit activity, MUA) are tightly related to hemodynamic signals. While LFPs in γ band are better in predicting hemodynamic signals elicited by short stimuli, MUA has better predictions to hemodynamic signals elicited by long stimuli. Our results also indicate that strong nonlinearity exists in neurovascular coupling.

## Introduction

Studies of neurovascular coupling explore the link between neural activity and hemodynamics [1], [2]. When neurons in the brain are firing more frequently, their demands for oxygen and glucose will increase [3]. Through certain signaling pathways, which are not fully understood [4], [5], the diameter of local arterioles expand and local blood supply will increase to meet the oxygen and glucose metabolic demands of increased neuronal activity. Functional imaging methods, such as fMRI [6], [7] and optical imaging [8], [9], identify such changes in blood supply (hemodynamics) to local brain regions in addition to the increased firing rate of neurons. Indeed, the identification of the relationship between hemodynamics and neural activity has been an important topic in neuroscience [7]. Although a linear neurovascular coupling system conceivably allows for the extraction of information about neural activity from hemodynamics since neural activity can be deconvolved from hemodynamics with a hemodynamic response function (HRF), previous fMRI and optical studies provide mixed conclusions on the linearity of neurovascular coupling. Whereas some claim a linear relationship [2], [10], [11], [12], [13], others identify nonlinearity in neurovascular coupling [14], [15], [16], [17].

There is an ongoing debate on whether hemodynamics is more tightly related to synaptic activity (represented by local field potential, LFP) or action potentials (represented by multi-unit activity, MUA or single-unit activity, SUA). Whereas studies suggest that brain hemodynamics is directly related to the spiking of neurons [12], [18], [19], evidences also indicate that hemodynamics is related more tightly to synaptic activity [2], [20], [21]. Recent developments in the field of optogenetics have provided a chance to address these questions on neurovascular coupling [19], [22], [23]. With optogenetics, specific cell types can be transduced with excitatory or inhibitory opsins, or both, making it possible to precisely control the firing of the transduced cells on demand [24], [25]. Optogenetics can benefit the study of neurovascular coupling in two ways. Firstly, we can study the neurovascular coupling system as driven by a specific cell type. Lee et al. have observed a positive blood oxygenation level-dependent (BOLD) signal while optically stimulating channelrhodopsin-2 (ChR2)-expressing pyramidal cells in the motor cortex of adult rats with optogenetic functional MRI (ofMRI) [22]. Kahn et al. have reported that BOLD response can be summated in an approximately linear fashion while stimulating ChR2-expressing pyramidal cells in the sensory cortex [23]. But the neural response was not recorded simultaneously with hemodynamics and the relationship between neural activity and BOLD signal was not examined. Secondly, by blocking the glutamatergic synapses in the sensory cortex, the contribution of pyramidal cells to the neurovascular coupling system was examined [19]. Thus optogenetics provides a tool to reach the subsystem and components in the neurovascular coupling system.

In this study, the neurovascular coupling system, driven by ChR2-expressing pyramidal cells in primary motor cortex (M1) of anesthetized rats, was examined with the combination of optogenetics and optical intrinsic signal (OIS) imaging. The motor cortex was chosen since most traditional non-optogenetic methods have limitations in studying the neurovascular coupling system in the motor cortex. While continuous electrical stimulation can directly stimulate the motor cortex, the simultaneous measurement of neural activity is unavailable during the stimulation because of the strong electrical artifacts caused by the electrical currents. In addition, electrical stimulation activates all cell types without cell type specificity. Combining optogenetics and OIS, we sought to examine the neurovascular coupling system in the rodent M1. The viral vector AAV9-CaMKIIα::ChR2(H134R)-mCherry was injected into the M1 region of adult rats, thus allowing us to only target CaMKIIα-expressing principal neurons and not GABAergic or glial cells. The blue light (∼473 nm) eliciting neural response was simultaneously and synchronously recorded with hemodynamic response. The results confirm that the firing of pyramidal cells in the motor cortex can reliably elicit hemodynamics under multiple stimulating durations. Further examinations indicate that both MUA and LFP signals are tightly related to hemodynamics. We also found strong nonlinearity in the local neurovascular coupling system.

## Materials and Methods

### Ethics statement

All protocols and procedures have been approved by the University of Florida Institutional Animal Care and Use Committee.

### Animal preparation

Adult male Sprague-Dawley rats (n = 10) weighing from 225 g to 500 g were used in the experiments. Prior to the injection of viral vector, anesthesia was induced using 4% isoflurane mixed with 1.0 L/min oxygen and 0.1 ml of xylazine given subcutaneously. Inhalation anesthesia was maintained at 1.5% to 2% isoflurane mixed with 0.4 L/min oxygen during the surgery. The top of the animal's head was shaved and cleaned with alternating swipes of betadine and 70% ethanol. A midline incision was made to expose the landmark bregma so that a small hole (1 mm in diameter) could be drilled on the skull above M1 with a handheld drill. Eight rats were stereotaxically injected with 2 µl AAV9-CaMKII::ChR2-mCherry (a kind gift from Dr. Karl Deisseroth, Stanford University) using coordinates of +2.0 mm AP, +3.0 mm ML right hemisphere, -2.0 mm DV [26]. Two control rats were injected with saline instead of viral vector using the same coordinates. Injections were performed using a 10 µl Hamilton syringe and a 34-gauge needle at a flow rate of 0.1 µl/min with an injection pump. After the injection, the needle was left in place for 5 minutes and then slowly removed. The scalp was sealed with wound clips and Bupivacaine was applied on the wound to manage pain. The animals were kept on a heating pad until they recovered from the anesthesia.

Experiments were performed between 3 and 5 weeks after viral vector injection to allow for adequate expression of ChR2 in pyramidal cells. Once anesthesia was induced (as described above), a midline incision was made so that the skull above motor cortex of both hemispheres (a region of around 5×10 mm^2^) could be removed to expose the brain surface. Once exposed, 1% agar and saline solution was used to cover the surface of the brain to protect it from drying. Anesthesia was maintained at 1.5% to 2% isoflurane mixed with 0.4 L/min oxygen throughout the experiments.

### Optical intrinsic signal imaging system

**Figure 1:**
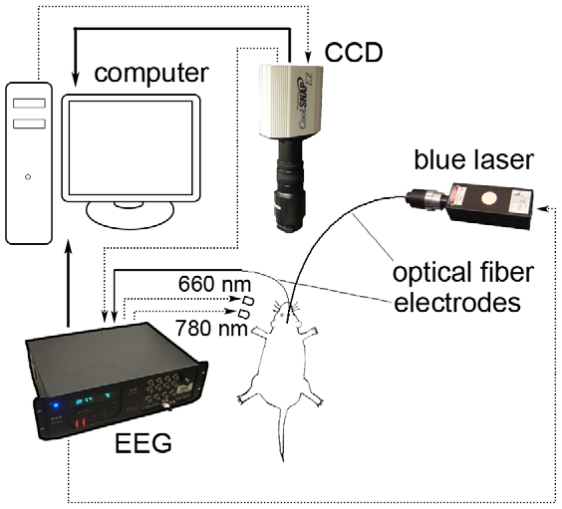
Diagram of the OIS system. Solid black arrows show the directions of data flows. Optical images are captured by the CCD camera and stored in the computer. Electrophysiological signals recorded by the electrodes are transferred to the EEG device and then stored in the computer. Dashed lines show the directions of control signals. The CCD camera sends a TTL signal to the EEG device while its shutter is open. Then the EEG device sends a 0.1 ms pulse to one of the LEDs. The two LEDs alternatively shine on the brain surface. The EEG device also sends 15 ms width pulses to turn on the blue laser. See Methods for a more detailed explanation of the system.

Our OIS system is schematically shown in Figure 1. The system uses two near-infrared (NIR) LEDs (780 nm and 660 nm, Epitex, SMB 780-1100-01-I, SMB 660N-1100-01) as light sources for optical imaging. The two LEDs alternatively illuminate the brain surface, while a digital CCD camera (CoolSNAP_EZ_, Photometrics Inc.) collects reflected light. Electrophysiological recording was performed by an EEG system (RZ5, Tucker-Davis Technologies, Inc.). A blue laser (473±10 nm, Shanghai laser & optics Co., Ltd.) was used as the light source for optogenetic stimulation. The whole system, including optical imaging, electrophysiological recording, and optogenetic stimulation, was synchronized by the RZ5 device. The CCD camera sent a TTL signal (1 ms width) to the RZ5 device while its shutter was open. The RZ5 device recorded the time of the TTL signal and sent out a 0.1 ms pulse to one of the two LEDs while the shutter of the camera was open. The CCD camera took 40 ms to capture and transfer one image to the computer. The sampling rate of hemodynamics was 12.5 Hz (80 ms duration) since two wavelengths were used. After the RZ5 device counted 250 TTL signals (10 s) from the CCD camera (which was 10 s baseline recording of hemodynamics), it began to send out 15 ms pulses to turn on the blue laser. To avoid interference between optical imaging and optical stimulation, we delivered one pulse of blue light (15 ms width) to the brain after the shutter of the CCD camera was closed. Thus our system can reliably image the hemodynamics even during the optical stimulation. The RZ5 device recorded the starting and ending time of electrophysiological recording. The RZ5 device also recorded the time when it sent out the signal to the LEDs and the blue laser. Thus, images captured by the CCD camera and the optical stimulation can be precisely aligned to the electrophysiological signal.

### Optogenetic stimulation

Optical stimulation was performed through an optical fiber (0.2 mm in diameter) coupled to the blue laser. The optical fiber was fed through an optrode for simultaneous recording and stimulation. The optrode consisted of four tungsten wires (50 µm in diameter) glued to a cannula (0.56 mm in outer diameter, 0.29 mm in inner diameter) with the tips of these wires deeper (by 0.4 mm) than the tip of the cannula to ensure illumination of the recorded neurons and avoid direct laser light on metal electrodes which can cause electrical artifacts that obscure LFP signals [27]. The continuous-wave (CW) energy density of the blue light at the optical fiber tip was approximately 180 mW/mm^2^. Six stimulating durations (0.6 s, 1 s, 2 s, 4 s, 12 s, and 24 s of blue light) with 15 ms pulse widths were performed. Fifteen trials were performed for each stimulating duration. The length of each trial was 120 s. Each trial contained a baseline of 10 s before the optogenetic stimulation began. Taking the stimulating duration of 600 pulses of blue light as an example, each trial contained 10 s of baseline, 24 s of stimulation and 86 s of recovery phase.

### Histology

**Figure 2:**
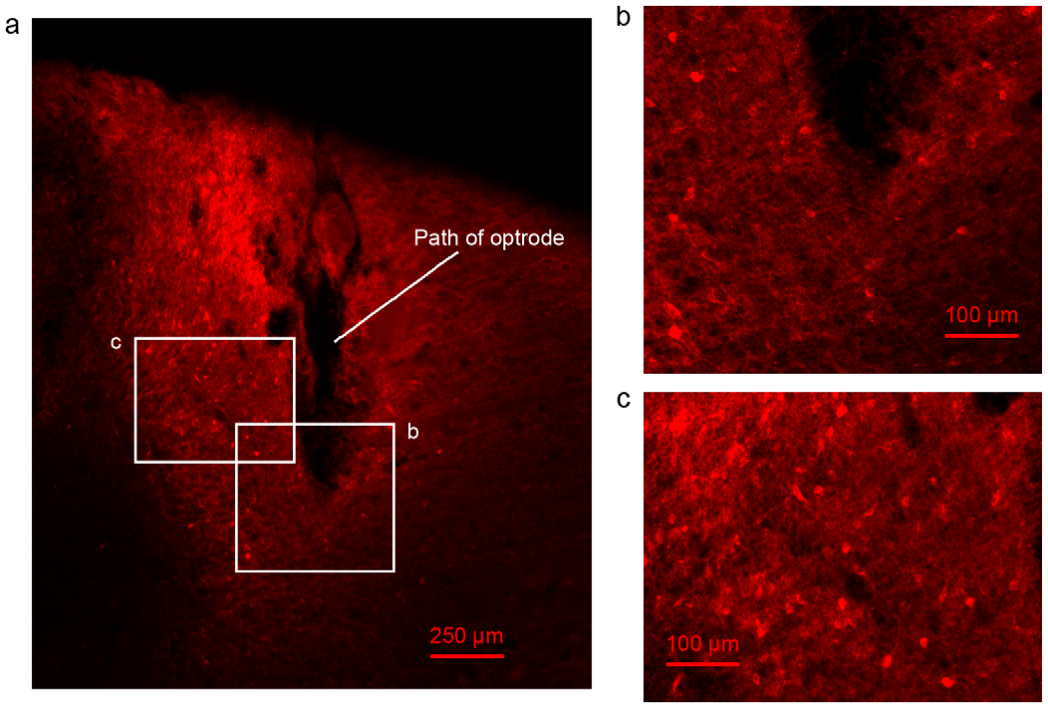
Expression of ChR2. **a.** A brain slice under fluorescent microscope with 4X magnification. The damaged region shows the path of the optrode. The bright red region shows the region with ChR2 expression. The bright red spots show the cell bodies with ChR2 expression. **b.** A region around the tip of optrode under fluorescent microscope with 10X magnification. The region is indicated by the white block b in **a**. **c.** A region along the path of the optrode under fluorescent microscope with 10X magnification. The region is indicated by the white block c in **a**. Cell bodies are clearly seen in **b**. and **c**.

To confirm the expression of ChR2, a perfusion-fixation was performed for all rats immediately after the cessation of experiments. Animals were deeply anesthetized with ketamine (70–100 mg/kg, IP) and isoflurane (4% mixed with 1.0 L/min oxygen). The animals were first transcardially perfused with saline and then 4% paraformaldehyde. The brain was extracted and fixed overnight in 4% paraformaldehyde, then cryoprotected in 30% sucrose in PBS. The brain was sectioned using a cryostat with a slice thickness of 50 µm. Microscopic fluorescence images were acquired to validate the gene expression, as shown in Figure 2. Figure 2A showed the fluorescence image under magnification of 4X. The damaged part of brain in Figure 2A showed the path of optrode. Two regions in Figure 2A were further enlarged under magnification of 10X to have a better view of cell bodies. One region around the tip of optrode was shown in Figure 2B. Another region along the path of optrode was shown in Figure 2C. It was clearly seen that there was very good ChR2 expression in the motor cortex.

### Electrophysiological recordings

**Figure 3:**
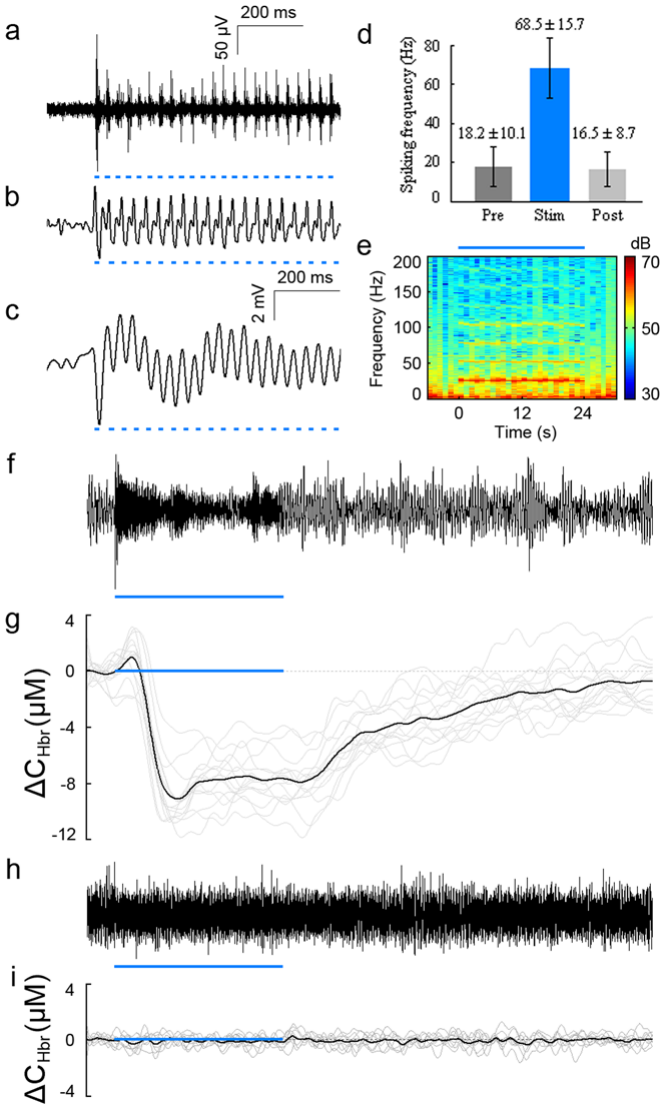
Neural and hemodynamic responses induced by optogenetic stimulation. **a.** MUA signal elicited by stimulation of ChR2-expressing pyramidal cells. Pulses of blue light are shown with short blue bars. **b**. LFP signal in γ band (30∼200 Hz) elicited by stimulation of ChR2-expressing pyramidal cells. **c**. LFP signal in the spectrum of lower than γ band (0.5∼30 Hz) elicited by stimulation of ChR2-expressing pyramidal cells. **d.** A significant increase in the firing rate of the MUA signal was observed during stimulation. A threshold of 5 SDs was set to the MUA signal. All spikes above the threshold were counted. **e**. The spectrum of LFP signal. The frequency range was from 0 to 200 Hz. The stimulating period was indicated by the blue bar. **f**. The broad-band EEG signal (0.5∼8K Hz) elicited by 24 s stimulation with ChR2 injected in M1. **g.** Hemodynamic response induced by 24 s stimulation with ChR2 injected in M1. The black line shows the average of 15 trials; the grey lines show hemodynamic responses of all 15 trials. **h**. The broad-band EEG signal (0.5∼8K Hz) elicited by 24 s stimulation with saline injected in M1. **i.** Hemodynamic response of control case with saline injected in M1. The black line is the average of 10 trials; grey lines show hemodynamic response of all 10 trials. The stimulating periods are indicated by the blue bars.

The optical fiber was inserted into the cannula of the optrode. The optrode was held by an electrode holder which was part of our stereotaxic device. Then the optical fiber and the optrode were stereotactically lowered into the brain so that the tip of the optical fiber and the tips of electrodes were 2 mm and 2.4 mm below the surface of the brain, respectively. The optrode and the CCD camera were carefully arranged so that the hemodynamic response was clearly seen in the CCD images. Electrophysiological recordings were performed at a sampling rate of 50K Hz. The measured electrophysiological signal was band-pass filtered between 600 Hz and 8K Hz to obtain the MUA signal (Fig. 3A). A threshold of 5 standard deviations was set to the MUA signal. The firing rates before, during and after the stimulation are shown in Figure 3D. The LFP signals in the γ band were obtained by band-pass filtering (30 to 200 Hz) of the electrophysiological signal (Fig. 3B). The LFP signals in the spectrum of lower than the γ band were obtained by band-pass filtering (0.5 to 30 Hz) of the electrophysiological signal (Fig. 3C). The spectrum of LFP signal was shown in Figure 3E.

### Calculation of Hbo and Hbr

A region of interest (ROI) was selected in the CCD images around the optrode where strongest hemodynamic response was observed. The ROI was a circle-shaped region with a radius of 0.2 mm. The time courses of optical signals at both 660 nm and 780 nm were averaged over the ROI to extract the optical signals at both wavelengths. The changes in concentrations of oxy-hemoglobin (Hbo) and deoxy-hemoglobin (Hbr) were resolved with modified Beer's Law [28]. If 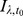 and 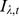 represent the optical signals acquired at baseline and during stimulation, respectively, the change in the absorption coefficient for the two wavelengths can then be calculated with the following equation:
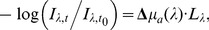
(1)in which 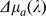 is the change of absorption coefficient for wavelength 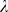. 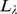 is the path length of photons in the brain tissue. In this work, we set 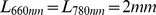 [29].

**Figure 4:**
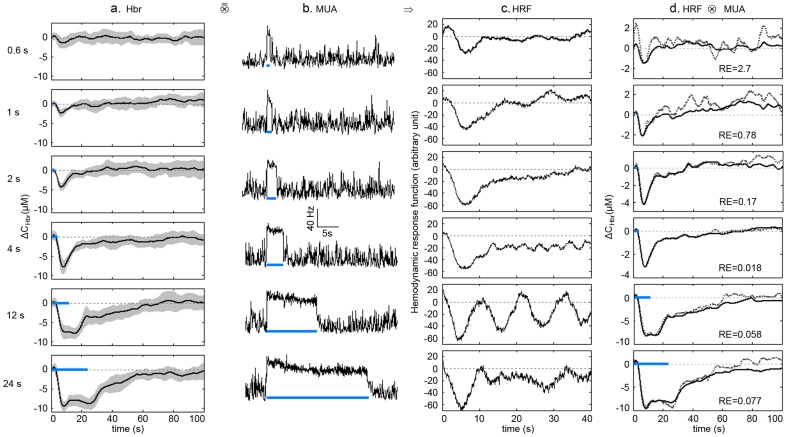
Predictions of Hbr with MUA signals. **a.** Hbr curves induced by stimulating durations of 0.6 s, 1 s, 2 s, 4 s, 12 s and 24 s of blue light. **b.** MUA responses (with the same sampling rate as the Hbr curves) for all the stimulating durations. **c.** HRFs generated with the Hbr curves and corresponding MUA signals with the MSE deconvolution method (see Methods for details). **d.** The measured (black solid lines) and predicted (grey dotted lines) Hbr curves. The predicted Hbr curves were generated by convolution of corresponding MUA signals in **b**. and HRFs in **c**. All Hbr and MUA curves were averaged from 15 trials. The blue bars indicate the stimulating periods. The “RE” in the figures shows the relative error (see methods for details) between predicted and the measured Hbr curves.

Since Hbo and Hbr are the major components associated with the changes in the absorption coefficients, the changes of concentrations of Hbo and Hbr can be calculated with following equation:
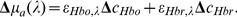
(2)where 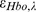 and 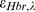 are the extinction coefficients for Hbo and Hbr, respectively, at 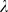. The values of 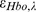 and 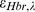 are known (e.g., from http://omlc.ogi.edu/spectra/hemoglobin/summary.html). Thus 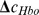 and 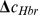 can be calculated with the measurements at two wavelengths. Only the Hbr signal was used in the following analysis since the Hbr signal was related to BOLD signal which was widely used in functional imaging [30]. The Hbr curves elicited by the 6 stimulating durations are shown in Figure 4A.

### Calculation of HRF

**Figure 5:**
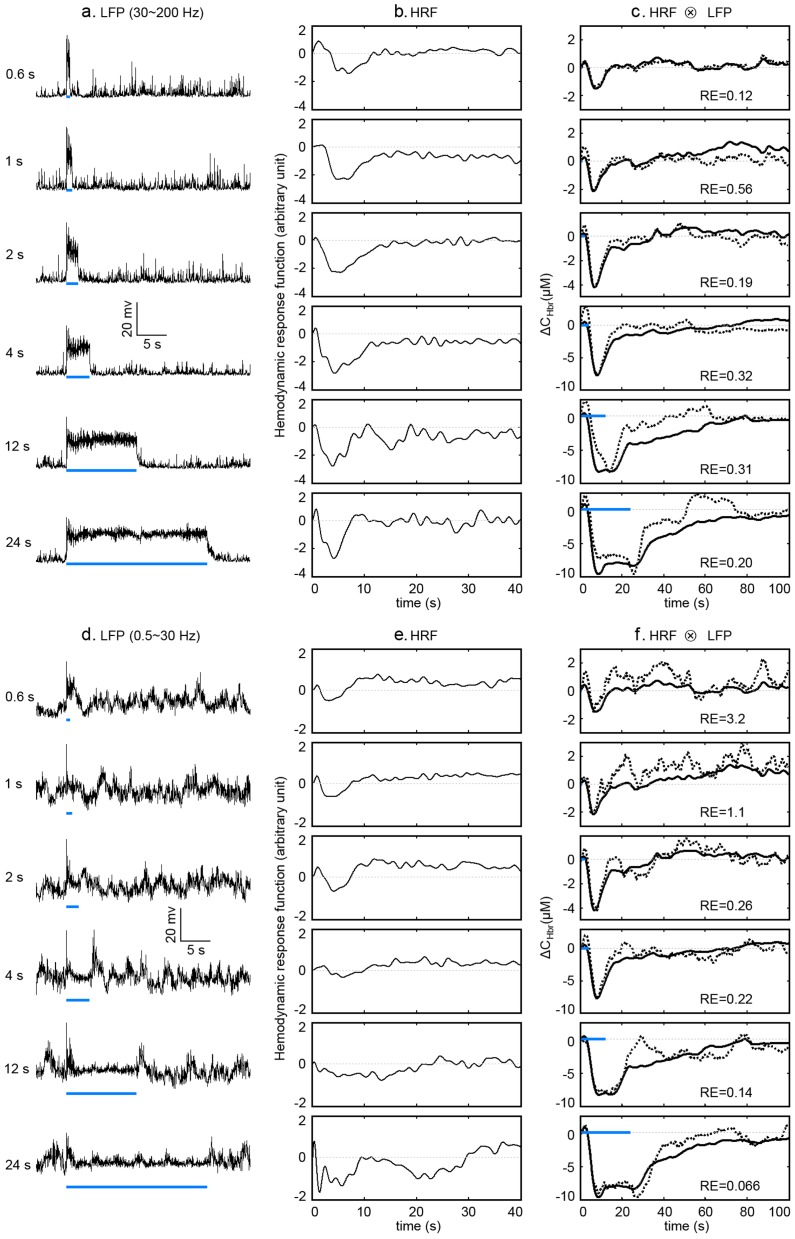
Predictions of Hbr with LFP signals. **a**. LFP responses in γ band for all the stimulating durations. The LFP signals were first band-filtered from 30 to 200 Hz. The absolute value of the LFP signals were then integrated with a time bin of 80 ms. **b**. HRFs generated with the Hbr curves (Fig. 4A) and corresponding LFP signals in γ band. **c**. The measured (black solid lines) and predicted (grey dotted lines) Hbr curves. The predicted Hbr curves were generated by convolution of corresponding LFP signals in **a**. and HRFs in **b**. **d**. LFP responses lower than γ band for all the stimulating durations. The LFP signals were first band-filtered from 0.5 to 30 Hz. The absolute value of the LFP signals were then integrated with a time bin of 80 ms. **e**. HRFs generated with the Hbr curves (Fig. 4A) and corresponding LFP signals in **d**. **f**. The measured (black solid lines) and predicted (grey dotted lines) Hbr curves. The predicted Hbr curves were generated by convolution of corresponding LFP signals in **d**. and HRFs in **e**. All Hbr and LFP curves were averaged from 15 trials. The blue bars indicate the stimulating periods. The “RE” in the figures shows the relative error (see methods for details) between predicted and the measured Hbr curves.

The integrated MUA and LFP signals were used in the linear convolution analysis. To obtain integrated MUA signals, all spikes above 5 standard deviations were detected and counted with a time bin of 80 ms to achieve the same sampling rate of the Hbr signal (Fig. 4B). To obtain integrated LFP signals, the absolute values of the LFP signals were integrated with a time bin of 80 ms to achieve the same sampling rate with the hemodynamic signal (Fig. 5A for γ band and Fig. 5D for bands lower than γ). Assuming the neurovascular coupling system is a linear system, the output (hemodynamics) can be produced by convolution of input (neural activity) and HRF:
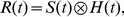
(3)where 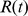 is the measured Hbr signal, 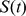 is the measured neural activity and 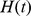 is the HRF. For discrete signals, the hemodynamic signal (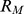), neural activity (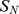) and HRF (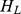) can be written as:
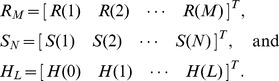
(4)


Thus Eq. 3 can be rewritten as:
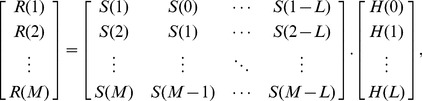
(5)or 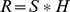. Thus the HRF can be estimated with the minimum square error (MSE) method:
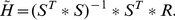
(6)


To use the MSE method to generate the HRF, it is important that 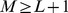. In this study, 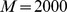 and 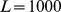
were used. It is also important to indicate that 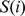 is the electrophysiological signal before the stimulation if 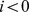.

### Summing-shifted-replicas method

Summing-shifted-replicas (SSR) method is used to predict the Hbr curves elicited by long stimuli using the Hbr curves of short stimuli [13]. Assume the duration of short stimulation is 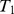, and the duration of long stimulation is 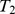 with 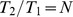. Then 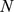 replicas of the Hbr curve elicited by short stimulation are used to predict the Hbr curve elicited by long stimulation. The first replica is not delayed, the second replica is delayed by 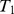, the third replica is delayed by 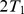, and so on. The 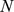 replicas of the Hbr curve elicited by short stimulation are summed up as the predicted Hbr curve elicited by long stimulation.

### Calculation of relative error

Let column vectors 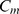 and 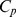 represent the measured and predicted Hbr curves, respectively. Then the relative error between the two curves is calculated with the following equation:
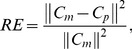
(7)where 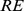 is the relative error, 
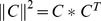
 with 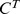 the transpose of 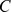.

## Results

### Neural and hemodynamic responses elicited by optogenetic stimulation

Optogenetic stimulation-induced neural responses were reliably observed at the stimulating site for all the stimulating durations. A representative MUA response is shown in Figure 3A. Every pulse of blue light has elicited reliable spiking of neurons. The firing rate of the MUA signal during stimulation is 68.5±15.7 Hz, which is much higher than the firing rates before (18.2±10.1 Hz) and after (16.5±8.7 Hz) the stimulation, as shown in Figure 3D. Representative LFP responses in the spectrum of γ band (from 30 to 200 Hz) and lower than γ band (from 0.5 to 30 Hz) are shown in Figures 3B and 3C, respectively. The amplitudes of LFP signals during stimulation are much higher than the amplitudes of baseline. Every pulse of blue light can elicit a significant change in the LFP signals. The spectrum of the LFP signal is shown in Figure 3E. Significant increase in power of LFP signal can be seen at the stimulating frequency (25 Hz) during the stimulating period.

The broad-band (from 0.5 to 8K Hz) EEG response with stimulation of 24 s is shown in Figure 3F. The dynamic change of Hbr elicited by stimulation of 24 s of blue light is shown in Figure 3G. Immediately following the stimulation, there was an increase in the concentration of Hbr, known as the “initial dip” [31], [32], [33], [34]. Interestingly, some previous studies observed the initial dip, while others did not [35], [36]. Here we report that OIS can reliably capture the initial dip induced by stimulation of cortical pyramidal cells. In addition, the initial dip can be seen with stimulation duration as short as 0.4 s (data not shown here). The Hbr curve elicited by stimulation of 24 s shows a peak-and-plateau pattern (Fig. 3G), which is also observed with electrical stimulation of a whisker [37]. The dynamic changes of Hbo and Hbr elicited by stimulation of 4 s are shown in Video S1. There was an increase in the concentration of Hbo and a decrease in the concentration of Hbr, which is similar to results found with traditional electrical stimulation [14]. No activation was observed for control rats injected with saline. The broad-band (from 0.5 to 8K Hz) EEG response with stimulation of 24 s and with saline injected in M1 is shown in Figure 3H. The resulting Hbr response is shown in Figure 3I. No obvious changes can be observed from Figures 3H and 3I. This indicates that the Hbr response shown in Figure 3G is elicited by optogenetic stimulation but not mechanical effect or other factors.

### Linear convolution analysis

We have used MUA, LFP in γ band (30∼200 Hz) and LFP lower than γ band (0.5∼30 Hz) to predict Hbr curves with linear convolution method. The dynamic changes of Hbr elicited by stimulating durations from 0.6 s to 24 s of blue light are shown in Figure 4A. Significant Hbr responses were observed for all stimuli. The integrated neural signals are shown in Figures 4B (MUA), 5A (LFP in γ band), and 5D (LFP lower than γ band). Significant increase in the firing rate of the MUA signal can be observed during the stimulation for all stimuli (Fig. 4B). Significant increase in the amplitude of LFP signal in γ band can also be clearly seen during the stimulation (Fig. 5A). However, increase in the amplitude of LFP signal lower than γ band is trivial and brief (Fig. 5D). HRFs are generated with the MSE deconvolution method (see Methods for details) with the Hbr signals and the corresponding integrated neural signals. The resulting HRFs are shown in Figures 4C, 5B, and 5E. The HRFs generated above are convoluted back with the corresponding integrated neural signals to obtain the predicted Hbr curves. The resulting predicted Hbr curves are shown as dashed lines in Figures 4D (MUA), 5C (LFP in γ band) and 5F (LFP lower than γ band), with the corresponding measured Hbr curves shown as solid lines.

Comparing the predicted curves in Figures 4D and 5C and 5F, it can be seen that MUA signals and LFP signals lower than γ band are better in predicting Hbr curves elicited by long stimuli, while LFP signals in γ band are better in predicting Hbr curves elicited by short stimuli. For stimulating duration of 0.6 s, predicted Hbr curve with LFP signals in γ band has much better agreement with measured Hbr curve (RE = 0.12, comparing to RE = 2.7 with MUA signal and RE = 3.2 with LFP lower than γ band). For stimulating durations of 1 s and 2 s, the predicted Hbr curves with MUA signals and LFP signals in γ band have comparable agreement with measured Hbr curves, while both are better than LFP signals lower than γ band. For stimulating durations from 4 s to 24 s, predicted Hbr curves with MUA signals have much better agreement with measured Hbr curves (RE values less than 0.1). In general, the quality of predictions with LFP signals in γ band is more uniform than that with MUA signals and LFP signals lower than γ band, which is indicated by the more uniform RE values. These results indicate that both LFP and MUA are tightly related to hemodynamic response. While LFP signals in γ band are better in predicting Hbr responses elicited by short stimuli, MUA signals are better in predicting Hbr responses elicited by long stimuli. Our results also indicate the nonlinearity of neurovascular coupling. None of MUA signals, LFP signals in γ band and LFP signals lower than γ band can achieve good predictions for all durations. Our results indicate that Hbr curves elicited by short stimuli can be linearly predicted with LFP signals in γ band, while Hbr curves elicited by long stimuli can be linearly predicted with MUA signals.

### A single HRF is not available for all stimuli

**Figure 6:**
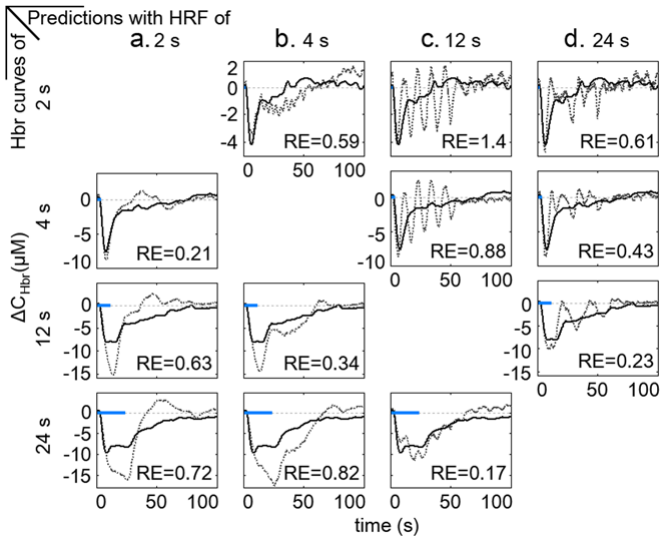
Cross-predictions. **a.** Measured Hbr curves elicited by 4 s, 12 s, and 24 s stimuli (black solid lines) and corresponding predicted Hbr curves with HRF of 2 s stimuli. **b–d.** Predictions with HRF of 4 s (**b.**), 12 s (**c.**) and 24 s (**d.**) stimuli. The blue bars indicate the stimulating period. Measured and predicted curves are shown in black solid lines and grey dotted lines, respectively. The “RE” in the figures shows the relative error between the predicted and the measured Hbr curves.

We have shown that Hbr curves elicited by long stimuli can be well predicted with HRFs generated from the Hbr curves and corresponding MUA signal. Therefore, it is important to know whether a single HRF is available to predict all Hbr curves elicited by long stimuli. HRFs generated from 2 s to 24 s of stimuli were used to cross-predict the Hbr curves elicited by durations from 2 s to 24 s of stimuli (Fig. 6, dashed lines, with the measured Hbr curves shown as solid lines). The predicted Hbr curves convoluted with HRF of short duration and MUA signal of longer durations have larger amplitudes than the measured Hbr curves, as can be seen from the lower-left part of Figure 6. In contrast, the predicted Hbr curves convoluted with HRF of long durations and MUA signal of shorter durations have smaller amplitudes than the measured Hbr curves, as shown in the upper-right corner of Figure 6. Furthermore, the predicted Hbr curves convoluted with HRF from 12 s and 24 s of stimuli and the MUA signals of shorter stimuli have a tendency of oscillating, as can be seen in Figure 6C–D. These poor predictions are also confirmed by the fact that the maximum relative error of any column is larger than 60%. These results indicate that the HRF perfect for one duration is not suitable for other durations. There is not a single HRF that can predict the Hbr curves of all durations.

### Prediction of Hbr curves with Hbr curves of shorter stimuli

**Figure 7:**
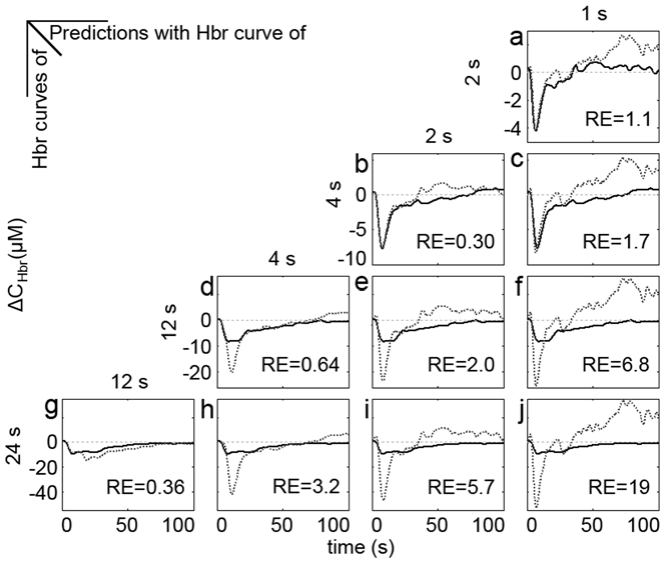
Prediction of Hbr curves with the summing-shifted-replicas method. **a.** Prediction of Hbr curve elicited by stimulation of 2 s with Hbr curve elicited by stimulation of 1 s. **b–c.** Predictions of Hbr curve elicited by stimulation of 4 s with Hbr curves elicited by stimulation of 2 s (**b.**) and 1 s (**c.**). **d–f.** Predictions of Hbr curve elicited by stimulation of 12 s with Hbr curves elicited by 4 s (**d.**), 2 s (**e.**) and 1 s (**f.**). **g–j.** Predictions of Hbr curve elicited by stimulation of 24 s with Hbr curves elicited by stimuli of 12 s (**g.**), 4 s (**h.**), 2 s (**i.**) and 1 s (**j.**). Measured and predicted curves are shown in black solid lines and grey dotted lines, respectively. The “RE” in the figures shows the relative error between the predicted and the measured Hbr curves.

We also examined the linearity of neurovascular coupling with the SSR method. The SSR method can avoid the possible mismatch between the electrophysiological recordings and the hemodynamics, i.e., the electrode samples a very small volume of brain tissue while the hemodynamics usually studies a much broader area. The SSR method is equivalent to a linear convolution model in which the Hbr curves elicited by short stimuli are viewed as the HRFs, while a step function serves as the stimulation. Hbr curves elicited by stimuli of 1 s, 2 s, 4 s, 12 s, and 24 s were used in the SSR analysis, allowing all longer durations to contain integer replicas of any shorter duration. The predictions were most accurate when the short and long Hbr curves were adjacent to each other (Fig. 7, dashed and solid lines represent predicted and measured Hbr curves, respectively). However, the predictions of Hbr curves elicited by stimuli of 12 s and 24 s exhibit much larger amplitudes than measured Hbr curves (Fig. 7, last two rows). Although the predictions shown in the first two rows of Figure 7 have comparable amplitudes with measured Hbr curves, the recovery phases greatly deviate from the measured curves. The poor predictions are also confirmed by the large relative errors (≥30% in all cases). These results indicate that Hbr curves of short stimuli cannot be used to predict Hbr curves of long stimuli with the SSR method.

## Discussion

This study investigates the neurovascular coupling system driven by cortical pyramidal neurons in the M1 area of adult rats. Cortical pyramidal cells were transduced with AAV9-driven ChR2 under a CamKII promoter and activated by blue light (∼473 nm) stimulation. Electrophysiological recordings and optical imaging were performed simultaneously and synchronously under varying stimulating durations. The coupling between the MUA signal, LFP in γ band, LFP lower than γ band and the Hbr signal was examined. We have shown that Hbr signals elicited by long stimuli (from 1 s to 24 s of blue light) can be well represented by a linear convolution model with MUA signal; while Hbr signals elicited by short stimuli (0.6 s and 1 s of blue light) can be well predicted with the LFP signal in γ band. These results indicate that both MUA and LFP signal are tightly related to hemodynamic responses. On the other hand, none of the neural signals (MUA, LFP in γ band and LFP lower than γ band) can well predict all durations. We have also shown that a single HRF does not exist for predicting the Hbr curves of all durations. Furthermore, the predictions are poor when Hbr curves elicited by short stimuli are used to predict the Hbr curves elicited by longer stimuli. These results indicate the strong nonlinear nature of neurovascular coupling.

It should be noted that optogenetic stimulation is different from traditional peripheral nerve stimulation in many ways. In peripheral nerve stimulation, the neural activities in cortex (both LFP and spiking) are driven by synapses. However, in optogenetic stimulation, synapses may or may not have a role in the generation of LFP and spiking signals. Furthermore, optogenetic stimulation of ChR2-expressing neurons generates a stimulation-related LFP signal, which is due to the current through the light-sensitive protein. The impact of these differences on the local neurovascular coupling system is still unknown. On the other hand, it should also be pointed out that optogenetic stimulation provides more options in the study of neurovascular coupling. By expressing ChR2 in up-streaming brain regions, it is possible to mimic traditional synapse-driven neurovascular coupling with optogenetic stimulation. Moreover, optogenetic stimulation offers the option of by-passing synapses to study neurovascular coupling system by blocking the local synapses [19].

It has been shown that artifacts may present in LFP signals while metal electrodes are used to record neural activity elicited by optogenetic stimulation [27]. We have minimized the artifacts by better arrangement of electrodes. As can be seen in Figures 3B and 3C, no significant artifacts are observed in the LFP signals recorded. The broad-band EEG signals shown in Figures 3F and 3H also indicate that the artifacts are trivial.

Many factors may influence the linearity of the neurovascular coupling system, while the mechanisms behind the coupling are still poorly understood. Products of metabolism, such as nitric oxide (NO) and adenosine, are believed to play a role in the expansion of arterioles [4]. It is reasonable to believe that the concentration of these products of metabolism must be high enough to expand the arteriole. For short stimuli, these products of metabolism might be largely diluted before reaching the receptors on the blood vessels. However, for long stimuli, the concentration of these products may reach a higher level. Thus a notable difference may be present between short and long stimuli. There is evidence that interneurons play a role in the neurovascular coupling [5]. However, during brief stimulation, the pathway through interneurons is not necessarily excited, due to the efficiency of synapses between pyramidal cells and interneurons. In summary, the nonlinearity of neurovascular coupling may exist in many aspects, which are still poorly understood.

Divergent conclusions have been made on the linearity of neurovascular coupling regarding the durations of stimuli. Considerable fMRI studies on humans showed that neurovascular coupling is nonlinear for short durations but linear for long durations [38], [39], [40]. However, opposite results were reported on animal models [37]. Here we show the neurovascular coupling system in the motor cortex of rats as nonlinear for short stimuli but linear for long stimuli. This suggests that basic neurovascular coupling unit may be consistent across species.

The results of this paper suggest that the local neurovascular coupling system is a duration-variant system. For long stimuli, the neurovascular coupling system remains a linear system. But the HRFs vary with different durations. This suggests that the structure of the local neurovascular coupling system is changing with different durations. However, the structure of the system does not change much for adjacent durations. For example, the HRF for stimuli of 2 s of blue light is a relatively good predictor of the Hbr curve elicited by 4 s of blue light (see the second rows in the third column of Fig. 6). The HRFs for durations of 12 s and 24 s of blue light are relatively good predictors of each other (the last two rows in the last two columns of Fig. 6). These observations indicate that only considerable changes in stimulating duration will appreciably vary the neurovascular coupling system.

Our results have indicated that the initial dip in hemodynamics is related to firing of cortical pyramidal neurons. The initial dip was reliably captured with the high spatial and temporal resolutions of our imaging system. Since the initial dip is more localized than the major increase of CBF [31], [32], the ability to reliably image the initial dip makes our system a promising tool for studying cortical functions at an early stage after the stimulation.

Neurovascular coupling is a complex dynamic system. Several types of neurons, such as primary pyramidal neurons, interneurons and astrocytes, may have a role in such a system. In this paper, we have studied the local neurovascular coupling system driven by the firing of local primary pyramidal neurons. This does not exclude the downstream participation of other types of neurons, but does elucidate the causal role of pyramidal forces in driving this dynamic. Unraveling different components in the neurovascular coupling system is another important task, which falls out of the scope of the current study.

## Supporting Information

Video S1 **Hemodynamics elicited by 4 s stimulation.** The imaging region is selected near the stimulating site. The left and right images in the upper part show the dynamic changes of Hbo and Hbr, respectively. The curves in the lower part are averaged over all pixels in the imaging region.
(MOV)
